# Predictors and Consequences of Homelessness: Protocol for a Cohort Study Design Using Linked Routine Data

**DOI:** 10.2196/42404

**Published:** 2023-07-27

**Authors:** Eileen Mitchell, Dermot O’Reilly, Diarmuid O’Donovan, Declan Bradley

**Affiliations:** 1 Centre for Public Health Queen’s University Belfast United Kingdom

**Keywords:** administrative data, data linkage, health care use, homelessness, housing, mortality

## Abstract

**Background:**

Homelessness is a global burden, estimated to impact more than 100 million people worldwide. Individuals and families experiencing homelessness are more likely to have poorer physical and mental health than the general population. Administrative data is being increasingly used in homelessness research.

**Objective:**

The objective of this study is to combine administrative health care data and social housing data to better understand the consequences and predictors associated with being homeless.

**Methods:**

We will be linking health and social care administrative databases from Northern Ireland, United Kingdom. We will conduct descriptive analyses to examine trends in homelessness and investigate risk factors for key outcomes.

**Results:**

The results of our analyses will be shared with stakeholders, reported at conferences and in academic journals, and summarized in policy briefing notes for policymakers.

**Conclusions:**

This study will aim to identify predictors and consequences of homelessness in Northern Ireland using linked housing, health, and social care data. The findings of this study will examine trends and outcomes in this vulnerable population using routinely collected health and social care administrative data.

**International Registered Report Identifier (IRRID):**

DERR1-10.2196/42404

## Introduction

### Overview

Worldwide, it is estimated that more than 100 million people are homeless [1]. Determining an accurate picture of the scale and impact of homelessness is challenging given the diverse definitions of homelessness that are used within and between countries [2-5]. The United Nations Office of the High Commissioner for Human Rights proposed that “experiencing homelessness means not having stable, safe, and adequate housing, nor the means and ability of obtaining it” [6]. The European Typology of Homelessness and Housing Exclusion framework explicitly categorized the different elements of housing insecurity and defined homelessness as “living in a place of habitation (during the reference period) that is below a minimum adequacy standard; and lacking access to adequate housing” [7].

The state of experiencing homelessness results from a complex range of connected factors, including social and economic contexts and policies [8]; cyclical housing markets, during which renters experience rent inflation during the upswing and risk eviction during a downturn; and the availability and quality of housing [9]. Within a society, there are factors that predict which individuals are at greatest risk of becoming homeless. Experiences of physical abuse, foster care experiences, incarceration, suicide attempts, and psychiatric problems, especially drug use problems, have been reported to be associated with an increased risk of becoming homeless in a systematic review and meta-analysis [10]. Taken together, the allocation of housing can be conceived as a complex queueing process that is stochastic on both the demand side (individuals in need of housing) and the supply side (when housing becomes available [11]).

The state of being homeless is important because people who experience it can be at increased risk of adverse outcomes compared to their peers who do not, especially those experiencing long-term and repeated homelessness [12,13]. Individuals who experience homelessness may have complex health care needs [14-18]. Homeless individuals are at greater risk of death, hospital admission related to substance use, and poor mental and physical health [14-16,19,20].

In the specific context of Northern Ireland, homelessness is a growing health and social issue [20-23], exacerbated by Northern Ireland’s history of conflict [24] and the current cost of living crisis [25]. A recent study found that 18 people were observed to be sleeping rough in Northern Ireland’s most recent survey in autumn 2020 [26], which was fewer than in previous years, likely due to service changes during the COVID-19 pandemic. The incidence of statutory homelessness in Northern Ireland cannot be easily compared to the rest of the United Kingdom because of the use of different definitions [7,27,28], but the Department for Communities reported 4306 households presenting as homeless between January and March 2021, of which 2717 were accepted as homeless (or threatened with homelessness), in priority need, and unintentionally homeless. The most common reason for homelessness (27%) was “accommodation not reasonable,” and for three-quarters of these, the underlying reason was physical health, disability, or mental health [29]. Homelessness among young adults is a growing public health concern in Northern Ireland [30]. Current figures estimate that around 3824 young adults, male and female, aged between 18 and 25 years presented as homeless to the Northern Ireland Housing Executive (NIHE) between 2018 and 2019 [31]. Over the past decade in Northern Ireland, there has been interest in using children’s social care data [21,31-33] to better understand the factors associated with young people becoming homeless. Social services may have a duty to accommodate children where they are without parents or if the parents cannot obtain long-term accommodation through a homeless application, for example, due to problems with their immigration status. There is limited information about how frequently children who experienced homelessness had repeated contact with child and family social services over time, through rereferrals, reregistration on the child protection register, and readmission to care rates, and how this is associated with deprivation and other child, family, and case characteristics.

People who experience homelessness are among the most deprived in society, may be underrepresented in government statistics, and are largely omitted from household surveys [23,24,34], making it difficult for researchers and policy makers to identify and address the needs of this vulnerable population group [23]. Administrative linkage data, once linked and appropriately analyzed, can be a useful source of insight to help policymakers with the consequences of homelessness and the policy interventions that might reduce harm and support health and well-being [35-37]. Information about the relationship between homelessness and health is limited in Northern Ireland [21-23,29,38]. Anonymized, linked health and social care (HSC) administrative data sets have considerable potential for measuring outcomes for individuals who are homeless. Linkage of data sets is powerful for conducting large-scale, longitudinal epidemiological research. Studies have also used emergency department (ED) administrative data to track trends and health inequalities and examined outcomes associated with being homelessness [39-41].

Linking homelessness records in Northern Ireland to HSC data sets and analyzing the data with epidemiological and reproducible modern data science methods will create knowledge with practical application in Northern Ireland, which will also be of interest to an international audience [32]. A careful study design is required to measure the effects of homelessness because of potential confounding between risk factors for and consequences of homelessness [42]. We aim to measure the scale of the issue of homelessness and its trends by place and over time, to understand what factors affect the risk of homelessness, and to understand the consequences of homelessness. This protocol describes our analysis plan to achieve this.

### Research Aims and Objectives

The aim of this work is to provide information for policy makers, practitioners, and the public about the causes and consequences of homelessness in Northern Ireland using linked health care and administrative data.

To achieve this, we seek to do the following:

1. Measure the incidence, prevalence, and duration of homelessness over time and place, overall, and in subgroup analysis by the reason for homelessness.
2. Describe the characteristics of people who have been homeless, overall and in subgroup analysis by the reason for homelessness, compared to the Northern Ireland population.
3. Investigate the risk factors for becoming homeless, based on personal characteristics, the time period, geographical factors, health-related factors, and social factors (eg, being a looked-after child).
4. Investigate the association between homelessness and health and social outcomes, including physical and mental health, and the use of HSC services.

## Methods

### Study Design 

The primary study design is a retrospective cohort study with nested matched cohort analysis. Homelessness will be defined as individuals registered as homeless with NIHE. Controls will be selected through propensity score matching [43] to the cases based on age, sex, geographical region, year and month of homelessness application, and a propensity score quantile from a multivariable logistic regression model. If assumptions are met, we will conduct a self-controlled case series study [44] as a sensitivity analysis.

### Study Population

We will access and analyze national data from Northern Ireland (approximately 1.9 million people). Individuals in Northern Ireland HSC (equivalent to the National Health Services of the rest of the United Kingdom) have a health and care number (H&C number), which is a unique identifier that is used across HSC in Northern Ireland. We will include individuals who were alive, had an H&C number, and had been registered with a general practitioner in Northern Ireland since 2010.

### Data Sources

The study will use administrative data obtained from several database sources, including the National Health Authority Information System (NHAIS), Electronic Prescribing Database (EPD), NIHE homelessness applications, patient administration system (PAS), ED information systems, Northern Ireland Maternity Information System (NIMATS), the Social Services Care Administrative and Records Environment (SOSCARE), and Pillar 1 and Pillar 2 COVID-19 data sets. The NHAIS contains information on all patients registered with a primary care physician. Northern Ireland has a universal, tax-financed, free at the point of service health care system with almost 100% population registration. It includes a unique address identifier (unique property reference number; UPRN). A list of temporary accommodations for people experiencing homelessness was compiled from stakeholder organizations and will be used to assign a binary indicator of whether an individual was registered as resident in such accommodation and the date registered to that address. NHAIS receives regular updates on the date and cause of death from the general register’s office. International Classification of Diseases, Tenth Revision (ICD-10) codes on death records derive from diagnoses recorded by the certifying the doctor on the death certificate. The PAS data set will be used to collate data relating to admitted patient care delivered by HSC hospitals in Northern Ireland, generated by the PASs within each hospital. These data are held centrally by the HSC Regional Data Warehouse. The SOSCARE data set will be used to collate data regarding information on children registered with social care services, including family support services, children in need services, and the child protection register. The PAS will be used to collate data on general hospital admissions and COVID-19 hospital admissions. The NIMATS data set will contain maternity care data on women and their children. The EPD data set contains detailed information on prescriptions issued in Northern Ireland. The Pillar 1 data sets are extracted from the laboratory information systems in Northern Ireland hospitals on a daily basis into a central repository in the HSC Regional Data Warehouse. It contains details of COVID-19 antigen tests carried out in each of the hospital laboratories, including those processed on behalf of primary care, social care, and community settings. Pillar 2 data processed by NHS Digital and extracted for Northern Ireland residents are sent to the Northern Ireland HSC Regional Data Warehouse. The NIHE data set will contain social housing data for information reasons for applying to be considered homeless, type of accommodation, and previous accommodation and assessment outcome. Table 1 lists the data sets for linkage and potential predictors of homelessness that will be analyzed.

**Table 1:** Data items or variables and data sources.

Data item		Data set
**Patient characteristics and confounders**		
	Age	NHAIS^a^
	Gender	NHAIS
	Socioeconomic status	NHAIS
	Underlying medical condition	EPD^b^
	History of health care use (eg, general practitioner consultations and hospital admissions)	Admissions and discharge data set
**Outcomes**		
	Maternity outcomes	NIMATS^c^
	Hospital admission	Admissions and discharge data set
	Emergency department	HBS^d^
	Prescribed medications	EPD
	Type of settlement (eg, private home, care home, or social housing)	NHAIS
	Homeless applicant status	NIHE^e^
	Homeless presenting reason	NIHE
	History with social services	SOSCARE^f^
	Cause of death	NHAIS
	COVID-19–related death	NHAIS
	Laboratory confirmed SARS-CoV-2 infection	Pillar 1 and Pillar 2
	Secondary SARS-CoV-2 infection due to household transmission	Pillar 1 and Pillar 2

^a^NHAIS: National Health Authority Information System.

^b^EPD: Electronic Prescribing Database.

^c^NIMATS: Northern Ireland Maternity Information System.

^d^HBS: Honest Broker Service.

^e^NIHE: Northern Ireland Housing Executive.

^f^SOSCARE: Social Services Care Administrative and Records Environment.

### Deidentification and Linkage

Linkage will be conducted by the Honest Broker Service (HBS). The personal data transfer to the Business Services Organisation (BSO) will be managed by separating the direct identifiers (name, date of birth, and address) from the rest of the data set at its source in NIHE and sending the 2 parts of the data sets to different teams in BSO for processing. A trusted third-party linkage team will only see the identifiers but never the individual attribute data. The researchers only see the attribute data but not the identifiers. A simple placeholder ID will be used to allow the information to be joined to other data sets without the use of the personal identifiers. The data processing steps are (1) NIHE will transfer a table of direct identifiers only to a “trusted third party” (the Family Practitioner Service; FPS) within the BSO, which is managerially separate and on separate computer networks from the HBS in the BSO. The FPS will aim to match the identifiers in the NIHE and NHAIS data sets (and thus to H&C numbers) using deterministic and fuzzy matching. (2) NIHE will send the main data set without identifiers (but with the placeholder ID) to the HBS. (3) The HBS will prepare all of the other data sets required for the project, assign a pseudonymous study ID to them, and upload the pseudonymized data to the secure research platform. (4) HBS will share with FPS a table (linkage key) that links all H&C numbers in Northern Ireland to the study ID. (5) FPS will join the NIHE identifier list with the matched H&C numbers to the junction table to assign a study ID. (6) Both HBS and FPS will destroy the pseudonymization key. (7) FPS will send a table of the study ID for each individual in the NIHE data set to HBS. (8) HBS will join the study ID from the NIHE to the homelessness data set using the placeholder identifier (eg, row number). The NIHE data set will then have a fully anonymized study ID that can be linked to all the other data sets in the project. HBS will upload the anonymized NIHE data to the secure research platform. The links between the tables will then rely on deterministic linkage. We will use the UPRN, time points, NIMATS, SOSCARE, and information about household size from the NIHE database to create groups of individuals who are associated together as households.

### Study Outcomes

For this study, 2 analytic epidemiological studies will be conducted: (1) study 1 will analyze the risk factors for homelessness, while (2) study 2 will examine homelessness as a risk factor for health and health care-related outcomes.

### Primary and Secondary Outcomes

In study 1, the primary outcome of interest will be statutory homelessness. For the purpose of propensity score matching, becoming homeless (ie, meeting the NIHE threshold for homeless in the housing data) in the quarterly time period will be dichotomized into a binary variable (true or false). We will explore whether residence in temporary accommodation should be combined in a composite indicator after exploratory data analysis. For the Cox regression analysis (described below), the time from the study start date to the first episode of homelessness in the NIHE data set will be used as the primary outcome. Secondary analyses will be conducted by the established reasons for homelessness.

In study 2, the primary outcome of interest will be death. Secondary outcomes will examine ED attendances, emergency hospital admissions, emergency hospital admissions with COVID-19, and COVID-19 vaccination. All will be measured as time from homelessness onset (of the person who experienced homelessness or their matched pair) to the event.

### Statistical Methods

We will commence analysis by conducting descriptive analyses to visually inspect trends in homelessness, trends over time in demand, supply, waiting list size, and waiting times for social housing, and risk factors (hospitalizations and deaths), including by age group, sex, geographical location, and index base year. We will undertake univariable and multivariable logistic regression for the first episode of homelessness. This will be conducted in R using *glm(family = “binominal”).* We will use *glm.predict()* to produce propensity scores for matching. We will use the propensity score to match individuals who have experienced homelessness with controls who have not experienced homelessness. We will match 1:1 on propensity score, age, sex, geographic area, and pregnancy status. Matching will be conducted over quarterly time periods to assign controls who shared characteristics at the time of homelessness. This will be implemented in a loop or apply function in R. Matched variables will not be used as independent variables. We will also undertake 2 alternative analyses to support the primary analysis. First, we will use a Cox proportional hazards model to investigate the hazard ratio of homelessness on the outcomes (censoring at death). Second, if the assumptions can be met, we will conduct a self-controlled case series analysis, which will give an alternative approach to managing confounding, fitting a spline for age.

For study 2, a Cox proportional hazards model will be used, comparing the propensity-matched groups over time to the primary and secondary outcomes producing hazard ratios and 95% CIs.

All tests will be 2-tailed, and a *P*<.05 will be considered statistically significant. All analyses will be carried out using R-Studio. Statistical analysis code will be shared on GitHub alongside publications.

### Data Storage and Management

The HBS will carry out the data linkage and offer a secure environment for researchers to access and analyze the pseudonymized individual-level data. The NIHE housing data set will be provided to the BSO family practitioner unit without the H&C number identifier. This unit will undertake deterministic and fuzzy matching according to their normal protocols to assign a H&C number. We will describe in our report the fraction of records that could not be assigned a H&C number. The H&C number will be used to link individuals’ data, though it will be replaced with an anonymous study ID in the analysis data set. All data will be securely stored on the HBS systems, and access will be revoked at the end of the project. HBS will archive and later destroy data according to its disposal schedule.

### Dissemination and Data Sharing

To enhance reporting transparency, this study will be reported in accordance with the guidelines for the Reporting of Studies Conducted Using Observational Routinely Collected Data (RECORD), which is an extension of the Strengthening the Reporting of Observational Studies in Epidemiology (STROBE) statement. The results from this work will be published as a full-length, peer-reviewed manuscript and presented at participant events and to a representative subgroup of key stakeholders from the homeless sector in Northern Ireland.

### Dissemination Plan

Our findings will be presented at national and international conferences and submitted for peer-reviewed publication to inform policy, practice, and research about the causes and consequences of homelessness.

### Ethical Considerations

Ethical approval was granted by Queen’s University governance and HBS. This study was approved by the National Research Ethics Service (Integrated Research Application System project ID 299887, Research Ethics Committees reference 22/SC/0065). The study was also approved by the HBS governance board (project 073). Data access agreements between the NIHE and the HSC BSO were established to manage the transfer and privacy-preserving deidentification and linkage of data. The research team will not have access to human participant information that can directly identify participants (eg, name, identifier, date of birth, or small geographic-level data). Informed consent was not required for this project. The research team will follow the statistical disclosure policy of the HBS, guided by the statistical disclosure control handbook. All outputs will be checked by the HBS team before being released. The analysis of individual-level data takes place within a safe setting at HBS from which data cannot be removed. Any potentially disclosive findings (eg, cross-tabs with small cell counts that may allow participant identification) will not be permitted to leave the safe setting. 

## Results

This project is the results of a partnership grant between the Administrative Data Research Northern Ireland (ADR NI), HBS, and NIHE. The transdisciplinary, multi-institutional team brings wide-ranging technical and stakeholder management experience, as well as many years of health prevention and evidence-based public health policy knowledge. The results from this project will aim to identify predictors and consequences of homelessness in Northern Ireland for the first time using linked homelessness, social services, and health administrative data. The results of our analyses will be shared with stakeholders, reported at conferences and in academic journals, and summarized in policy briefing notes for policymakers. As of September 2023, initial data analysis is expected to commence on this project and continue until March 2026.

## Discussion

### Overview

Our planned study will analyze HSC administrative data to investigate the characteristics, exposures, and outcomes of people who experience homelessness. Research from the United Kingdom [45], Ireland [46], Scotland [47], and Canada [35] has elicited useful policy insights for those contexts at the time points at which they were conducted. Our work will add to the understanding of the relationship between homelessness and health, which is an important contemporary social justice concern [48].

The complex nature of homelessness and its interactions with health require a tailored study design to provide evidence about the direction of causality in associations. For example, ill health could conceivably lead to or result from experiencing homelessness. Our study design introduces a time component that will allow us to understand the order of events and an approach to controlling confounding that will allow us to separate the contribution to homelessness of many interrelated socioeconomic factors. People who reside in temporary accommodations may face challenges around crowding. The impacts of the pandemic on the incidence and prevalence of homelessness remain uncertain at the time of writing [49,50]. However, there is emerging global evidence of higher COVID-19 infection and death rates among individuals who experience homelessness [51-53].

Strengths of this study include the use of a large, whole-population cohort, which should have a relatively low risk of bias. We also have access to a range of routine, whole-population data sources. However, there are limitations to this study. First, our definition of homelessness is restricted to individuals registered as being homeless with the NIHE. This will not detect individuals who were homeless but did not seek housing from NIHE. We will explore the use of residence in temporary accommodations, but at this point in planning, we do not have evidence about the usefulness of this indicator, given that it would require individuals to update their health care registration address. Second, our study will be limited to the Northern Ireland health care context, and results may not be applicable to other settings. There is a risk of bias if individuals experiencing homelessness have a greater risk of health care records not containing their unique identifier.

### Conclusion

The findings of this study will be useful for researchers and policymakers seeking to study trends and outcomes associated with being homeless within a Northern Ireland context and will also be applicable to other contexts. Our study will provide evidence that can inform policymakers and highlight the potential societal benefits from the safe use of deidentified administrative data.

## Abbreviations

ADR NI: Administrative Data Research Northern Ireland
BSO: Business Service Organisation
ED: emergency department
EPD: Electronic Prescribing Database
FPS: Family Practitioner Service
H&C number: health care number
HBS: Honest Broker Service
HSC: health and social care
ICD-10: International Classification of Diseases, Tenth Revision
NHAIS: National Health Authority Information System
NIHE: Northern Ireland Housing Executive
NIMATS: Northern Ireland Maternity Information System
PAS: patient administration system
RECORD: Reporting of Studies Conducted Using Observational Routinely Collected Data
STROBE: Strengthening the Reporting of Observational Studies in Epidemiology
SOSCARE: Social Services Care Administrative and Records Environment
UPRN: Unique Property Reference Number

## Data Availability

The data sets generated during and/or analyzed during this study are not publicly available, due to being controlled by the HSC Regional Business Services Organisation (RBSO/BSO). Data is available from the Honest Broker Service within RBSO/BSO upon request.
